# Urinary Tract Infection among Antiretroviral Therapy Users and Nonusers in Jimma University Specialized Hospital, Jimma, Ethiopia

**DOI:** 10.1155/2014/968716

**Published:** 2014-04-16

**Authors:** Serkadis Debalke, Waqtola Cheneke, Haimanot Tassew, Mohammed Awol

**Affiliations:** Department of Medical Laboratory Sciences and Pathology, College of Public Health and Medical Sciences, Jimma University, Jimma, Ethiopia

## Abstract

*Background.* The introduction of antiretroviral therapy (ART) has dramatically reduced morbidity related with bacterial infection including urinary tract infection (UTI) among patients with HIV/AIDS. This study was carried out to determine the prevalence of UTI and identify common bacterial etiologies among HIV/AIDS patients of ART users and nonusers in Jimma University Specialized Hospital. *Methods.* A comparative cross sectional study was conducted from September to December 2012 on 367 ART users and 114 nonuser patients attending ART clinic. Sociodemographic characteristics, associated factors, and urine samples were collected; culture, biochemical tests, Gram stain, and drug sensitivity tests were done. * Results.* Of 467 examined urine samples, 56 (12%) had significant bacterial growth. Forty-six (12.5%) of the cases were ART users and 10 (10%) were nonusers. *E. coli* was the predominant isolate in both ART users (25 (54.3%)) and nonusers (6 (6%)). Majority of the bacterial isolates were from females. Most (>75%) of the isolates from both groups were resistant to ampicillin and trimethoprim-sulfamethoxazole but sensitive to norfloxacine, ceftriaxone, and chloramphenicol. * Conclusion*. There was no statistically significant difference regarding the prevalence of significant bacterial growth between ART users and nonusers. Therefore, it is recommended that UTI in both groups should be managed with drugs that show sensitivity.

## 1. Introduction

Globally, an estimated 34 million people are living with human immunodeficiency virus (HIV) with a high (1.9 million) number of newly infected people in Sub-Saharan Africa. Annually, an estimated 1.8 million people are dying of HIV/AIDS related diseases [1]. In people living with HIV/AIDS, almost every part of the genitourinary system is affected with different diseases [2]. In addition, such people are more vulnerable to different bacterial infections including urinary tract infection (UTI) because of high viral load and low CD4 count of the infected individuals [3]. Different researchers have shown an increased prevalence rate of UTI in HIV/AIDS patients: prevalence rate of 6.3%–41% was reported from various parts of the world [3–5].

Antiretroviral therapy (ART), however, improves the health of people infected with HIV/AIDS through decreasing the progression of the infection, restoration of the immunity of the patient, decreasing the viral load, and reducing the opportunistic infections [6–8]. Studies on the evaluation of the effect of highly active antiretroviral therapy (HAART) show that ART has a significant impact on reduction of the incidence of bacterial infections including bacteremia, bacterial pneumonia, and urinary tract infections that occur in HIV infected patients [9].

*Staphylococcus aureus (S. aureus) *is the predominant bacterial uropathogen amongART user patients [10, 11].* Escherichia coli (E. coli)* [3, 4],* Enterococcus* species,* Pseudomonas aeruginosa (P. aeruginosa)*,* Klebsiella*,* Acinetobacter*,* Proteus* species,* Candida*, and* Salmonella* species are also found among HIV infected patients [3–5].

As far as our knowledge to date is concerned, there is no published report on prevalence, etiologic agent, and the antimicrobial susceptibility pattern of bacterial uropathogens among ART user patients in this study setting. Thus, the current study was undertaken to determine the etiologic agent and antimicrobial susceptibility pattern of bacterial uropathogens among ART user patients in Jimma University Specialized Hospital.

## 2. Materials and Methods

### 2.1. Study Area and Population

A comparative cross-sectional study was carried out between September and December 2012 in Jimma University Specialized Hospital, Jimma town, South West Ethiopia. In the study, 367 ART users and 114 nonusers of HIV/AIDS patients were interviewed and their respective urine samples were collected. Patients with and without symptom of UTI who attended the ART Clinic during the study period were included. Patients who were on antibiotics, pregnant mothers, and those younger than 15 and older than 50 years were excluded.

### 2.2. Sample Size and Sampling Technique

The sample size was determined based on a predicted 41% prevalence of urinary tract infection among HIV positive patients, ±5% precision, and 95% confidence interval [5]. Accounting for a 10% nonresponse rate, the final sample size estimated was 481. Proportional allocation of the sample size was made for both ART user and nonuser groups. Patients were selected consecutively as they appeared at the ART Clinic for their regular followup.

### 2.3. Sample Collection and Processing

Data on sociodemographic characteristics and other associated variables were collected using structured, pretested questionnaire. Fresh midstream urine samples were collected from every study participant after they were oriented towards how to collect midstream urine. All urine samples were processed and cultured within an hour of collection. Samples, for which delay is unavoidable, were stored in refrigerator until processed. Those urine samples contaminated with feces were rejected. Each specimen was well-mixed using sterile calibrated wire loop with a diameter of 2 millimeter (holding 0.002 milliliter). The loop, full of urine, was inoculated onto MacConkey agar, mannitol salt agar, and nutrient agar and plates were incubated for 24 hours at 37°C. Isolates were considered significant if there were ≥10^5^ colony forming units/milliliter (CFU/mL). Bacterial identification was made using biochemical tests, namely, indole, citrate, Kligler iron agar (KIA), lysine decarboxylase, urea hydrolysis, catalase, coagulase, and mannitol fermentation [12].

Antimicrobial susceptibility pattern was determined using Kirby-Bauer's disk diffusion method following Clinical and Laboratory Standard Institute (CLSI) guidelines [12]. Bacterial isolates were tested for different types of routinely used antibiotics (7 for Gram negative and 8 for Gram positive bacteria). The antibiotic disks were ampicillin (AMP, 10 *μ*g), trimethoprim-sulfamethoxazole (SXT, 25 *μ*g), nalidixic acid (NA, 30 *μ*g), ceftriaxone (CRO, 30 *μ*g), chloramphenicol (C, 30 *μ*g), nitrofurantoin (F, 300 *μ*g), norfloxacin (NOR, 10 *μ*g), clindamycin (DA, 2 *μ*g), and erythromycin (E, 15 *μ*g). All the antimicrobials used for the study were obtained from Oxoid Ltd. Bashingstore Hampaire, UK.

A standard inoculum adjusted to 0.5 McFarland was swabbed onto Muller-Hinton agar (Oxoid Ltd. Bashingstore Hampaire, UK); antibiotic discs were dispensed after drying the plate for 3–5 minutes and incubated at 37°C for 16–18 hours. After incubation, the zones of growth inhibition were determined and then reported as sensitive and resistant by comparing the zone of inhibition with the standard table [12]. The reference strains used as control were* E. coli* (ATCC 25922) and* S. aureus* (ATCC 25923).

### 2.4. Data Analysis and Interpretation

Data entry and analysis were done using statistical package for social science (SPSS), version 16 software. The chi-squared test (*χ* ^2^) was used to determine the presence of statistically significant associations between the dependent variables and the independent variables. Statistical significance was considered at *P* value < 0.05.

### 2.5. Ethical Consideration

Ethical approval for this study was obtained from the Jimma University College of Public Health and Medical Sciences Research Ethical Clearance Board and from the Research Project Office of Jimma University. A written informed consent (translated to Amharic) was obtained from each participant before collecting the data. All the information was kept confidential throughout the study. The results of all patients were disclosed for the responsible person (nurses and physician) for prescriptions of drugs for those UTI positive patients.

## 3. Results

In this study, 481 HIV seropositive patients were enrolled. Of these, 367 were ART users and the rest 114 were nonusers. Fourteen samples of non-ART user patients were rejected due to contamination. Majority of the participants were females, 301 (64.5%), and the age ranged between 25 and 34 years, 220 (47%) (Table 2). From the total 467 urine samples examined, only 56 (12%) had significant bacterial growth. Forty-six (12.5%) of the cases with significant bacterial growth were observed in ART users and 10 (10%) were observed in non-ART user patients (*P* = 0.48) (Table 1).

Prevalence of UTI was significantly higher among female patients (44 (14.6%)) than male (12 (7.2%)) patients (*P* = 0.02). However, there was no statistically significant association with religion and marital status (*P* > 0.05) (Table 2).

Likewise, there was no statistically significant association between UTI and associated factors including previous history of UTI and prolonged antibiotic use (*P* > 0.05) (Table 3).

Ten different types of bacteria were isolated from urine culture of ART users whereas only four types of bacteria were isolated from non-ART users. Gram negative bacteria were the predominant isolates (49 (87.5%)) compared to Gram positive bacteria which comprise 7 (12.5%). In both ART users and nonusers,* E. coli* was the predominant bacteria which accounted for 25 (54.3%) and 6 (60%), respectively.* Enterobacter aerogenes* (*E. aerogenes*) (6 (13%)),* S. aureus* (3 (6.5%)),* Edwardsiella* species (2 (4.4%)),* Staphylococcus saprophyticus *(*S. saprophyticus*) (1 (2.2%)),* P. aeruginosa* (1 (2.2%)), and* Klebsiella pneumoniae *(*K. pneumoniae*) (1 (2.2%)) were only found in ART users (Table 4).

The results of antimicrobial susceptibility pattern for bacteria isolated from urine culture of ART and non-ART user patients are presented in Tables 5 and 6. Greater than 80% of Gram negative organisms isolated from ART users were sensitive to norfloxacin (82.5%), ceftriaxone (92.5%), chloramphenicol (100%), and nitrofurantoin (95%). Similarly, most of the isolates of non-ART user patients were sensitive to norfloxacin (66.7%), ceftriaxone (77.8%), chloramphenicol (88.9%), and nitrofurantoin (100%). However, more than 80 percent of Gram negative bacteria isolated from ART user patients were resistant to ampicillin (82.5%) and trimethoprim-sulfamethoxazole (87.5%). Likewise, isolates of non-ART users were resistant to these two drugs: ampicillin (88.9%) and trimethoprim-sulfamethoxazole (77.7%).

All Gram positive organisms isolated from ART users were 100% sensitive to norfloxacin, ceftriaxone, chloramphenicol, erythromycin, and clindamycin. * S. epidermidis* isolated from non-ART user patient was 100% sensitive to ceftriaxone, erythromycin, and clindamycin; however, it was 100% resistant to norfloxacin, chloramphenicol, ampicillin, nalidixic acid, and trimethoprim-sulfamethoxazole.

## 4. Discussion

The present study showed the distribution and antibiotic susceptibility pattern of bacteria isolated from ART and non-ART users among HIV positive patients in Jimma University Specialized Hospital. In this study, an overall prevalence rate of UTI among HIV/AIDS patients was 56 (12%) which was lower than the prevalence rate reported by Zagreb (41%) [5]. This difference could be due to the difference in the degree of the immune status of the patients who had participated in the studies which might contribute to an increase in the occurrence of UTI.

On the other hand, lower prevalence rate (6.3%) was reported from Nigeria [4]. This might be due to the difference in the disease stage among the participants. In the present study, stages III and IV AIDS patients were included who were not included in the previous study. Incidence of UTI is increased among AIDS patients than asymptomatic HIV infected people [13].

In the present study, there was no statistically significant difference (*P* = 0.48) observed on the magnitude of UTI among ART users (46 (12%)) compared to non-ART users (10 (10%)). The finding was not in line with a study conducted in Nigeria where prevalence of UTI was higher (25.3%) among ART users compared to the control groups (13%) [10]. Similarly, another study conducted in Nigeria showed significantly higher prevalence of UTI among HAART (27.78%) compared to the control groups (17.31%), though the control groups were non-HIV subjects [14]. On the other hand, significant reduction in UTI among HIV/AIDS patients using antiretroviral therapy was reported from Italy [9]. This might be due to other UTI related factors like genital hygiene practices, sexual activity, and others [14]. However, in this study data on such factors were not collected. Additional research is needed to better understand this condition.

Prevalence of UTI was significantly higher among female patients in both ART and non-ART user patients than male and the difference was statistically significant (*P* = 0.02). Similar findings were reported from Ethiopia [15, 16] and elsewhere in the world [4, 17]. This is probably because of the anatomy of the female genitourinary tract that is short and close to the anal and vaginal openings which facilitates the entry of the infective organisms to the urethra.

In our study, the types of bacterial etiologies associated with UTI were higher in ART users (10 species) as compared to non-ART users (4 species only). However, Gram negative bacteria were the most common isolate that accounted for 49 (87.5%) of all clinically significant urinary isolates.* E. coli* was the predominant isolate in both ART (60%) and non-ART (54%) user groups. This is comparable with earlier study done in Nigeria [4, 13, 17] among HIV/AIDS patients. However, according to another study done in Nigeria, Benin [11] and Calabar City [10], the predominant bacteria among ART users were* S. aureus* (87.2%) followed by* E. coli* 84% while* E. coli* was common among non-ART users [10]. The infecting organisms identified in this study are in agreement with commonly isolated bacteria in other studies of general population elsewhere in the world [18] and in Ethiopia [15, 19].

In the present study, more than 75% of Gram negative organisms isolated from ART users and nonusers were sensitive to norfloxacin, ceftriaxone, chloramphenicol, and nitrofurantoin while being resistant to ampicillin and trimethoprim-sulfamethoxazole. However, in a study conducted in Nigeria, Benin City, isolates were only sensitive to nitrofurantoin [11] and resistant to other antibacterial agents used (amoxicillin, cotrimoxazole, ciprofloxacin, and ofloxacin) [11]. In Calabar City, the organisms were highly resistant to the commonly used antibiotics such as chloramphenicol and cotrimoxazole but sensitive to ciprofloxacin, ofloxacin, sparfloxacin, and refloxacin [10]. These differences might be due to the irrational drug utilization habit of the communities or to the overdistribution of those sensitive and resistant strains of bacteria. In conclusion, the magnitude of UTI among ART and non-ART users in the present study was comparable; however, the type of bacteria among ART users was higher than non-ART users.* E. coli* was the most frequently isolated species in both ART user and nonuser patients. Most of the bacterial isolates from antiretroviral therapy user and nonuser patients were resistant to ampicillin and trimethoprim-sulfamethoxazole but sensitive to norfloxacin, ceftriaxone, and chloramphenicol.Therefore, it is recommended that urinary tract infection in both antiretroviral users and nonusers should be managed with those drugs that were found to be sensitive. Moreover, further study is recommended in order to understand the reason why there are more types of organism found in ART users than nonusers. Further study is also recommended to identify the reason for insignificant difference in prevalence rate between the two groups by considering the determination of CD4 cell count, viral load, ART drug adherence, and other such parameters.

## Figures and Tables

**Table 1:** Prevalence of urinary tract infection among ART and non-ART user patients, Jimma University Specialized Hospital, Ethiopia, 2012.

ART use	UTI infection		Total number (%)
	Positive number (%)	Negative number (%)	
Yes	46 (12.5)	321 (87.5)	367 (100)
No	10 (10)	90 (90)	100 (100)
Total	**56 (12)**	**411 (88)**	**467 (100)**

**Table 2:** Prevalence of UTI by sociodemographic features among seropositive ART user and non-ART user patients, JUSH, Ethiopia, 2012.

Characteristics	Examined number	Infected number (%)
Age		
15–24	47	4 (8.5)
25–34	220	32 (14.5)
>34	200	20 (10)
Sex		
Male	166	12 (7.2)
Female	301	4414.6
Religion		
Christian	302	37 (12.3)
Muslim	165	19 (11.5)
Marital status		
Single	76	7 (9.2)
Married	253	33 (13)
Widowed	55	4 (7.3)
Divorced	83	12 (14.5)
Educational level		
Illiterate	80	12 (15)
Primary (1–8)	230	28 (12.2)
Secondary (9–12)	122	13 (10.7)
Tertiary (>12)	35	3 (8.6)

**Table 3:** Prevalence of UTI by associated factors among seropositive ART user and non-ART user patients, JUSH, Ethiopia, 2012.

Characteristics	Examined number	Infected number (%)
Previous history of UTI		
Yes	249	29 (11.6)
No	218	27 (12.4)
Prolonged antibiotic use		
Yes	116	12 (10.3)
No	351	44 (12.5)
Clinical case		
Symptomatic	146	20 (13.7)
Asymptomatic	321	36 (11.2)

**Table 4:** Distribution of etiologic agents of UTI among ART and non-ART user patients, Jimma University Specialized Hospital, Ethiopia, 2012.

Etiologic agent	Patients		Total number (%)
	ART user number (%)	Non-ART user number (%)	
*E. coli *	**25 (54.3)**	**6 (60)**	**31 (55.4)**
*E. aerogenes *	**6 (13)**	**0 (0)**	**6 (10.7)**
*K. rhinose *	**2 (4.4) **	**2 (20)**	**4 (7.1)**
*P. alcalifaciens *	3 (6.5)	1 (10)	**4 (7.1)**
*S. aureus *	3 (6.5)	0 (0)	**3 (5.4)**
*S. epidermidis *	2 (4.4)	1 (10)	**3 (5.4)**
*Edwardsiella* spp.	2 (4.4)	0 (0)	**2 (3.6)**
*S. saprophyticus *	1 (2.2)	0 (0)	**1 (1.8)**
*P. aeruginosa *	1 (2.2)	0 (0)	**1 (1.8)**
*K. pneumoniae *	1 (2.2)	0 (0)	**1 (1.8)**
Total	**46 (100)**	**10 (100)**	**56 (100)**

**Table 5:** Antimicrobial sensitivity pattern of Gram negative bacteria isolated from urine culture of ART and non-ART user patients, Jimma University Specialized Hospital, Ethiopia, 2012.

Isolated bacteria	Sensitivity pattern	Antimicrobial agent number (%)						
		NOR	C	CRO	AMP	NA	F	SXT
ART user								
*E. coli* (*n* = 25)	S	21 (84)	24 (96)	25 (100)	3 (12)	12 (48)	25 (100)	1 (4)
	R	4 (16)	1 (4)	0 (0)	22 (88)	13 (52)	0 (0)	24 (96)
*E. aerogenes* (*n* = 6)	S	5 (83.3)	6 (100)	6 (100)	2 (33.3)	4 (66.7)	6 (100)	0 (0)
	R	1 (16.7	0 (0)	0 (0)	4 (66.7)	2 (33.3)	0 (0)	6 (100)
*K. rhinose * (*n* = 2)	S	1 (50)	2 (100)	2 (100)	1 (50)	1 (50)	2 (100)	0 (0)
	R	1 (50)	0 (0)	0 (0)	1 (50)	1 (50)	0 (0)	2 (100)
*P. alcalifaciens* (*n* = 3)	S	2 (66.7)	1 (33.7)	3 (100)	0 (0)	0 (0)	2 (66.7)	1 (33.3)
	R	1 (33.3)	2 (66.7)	0 (0)	3 (100)	3 (100)	1 (33.3)	2 (66.7)
*Edwardsiella* spp. (*n* = 2)	S	2 (100)	2 (100)	2 (100)	0 (0)	2 (100)	2 (100)	1 (50)
	R	0 (0)	0 (0)	0 (0)	2 (100)	0 (0)	0 (0)	1 (50)
*P. aeruginosa* (*n* = 1)	S	1 (100)	1 (100)	1 (100)	1 (100)	1 (100)	0 (0)	1 (100)
	R	0 (0)	0 (0)	0 (0)	0 (0)	0 (0)	1 (100)	0 (0)
*K. pneumoniae* (*n* = 1)	S	1 (100)	1 (100)	1 (100)	0 (0)	1 (100)	1 (100)	1 (100)
	R	0 (0)	0 (0)	0 (0)	1 (100)	0 (0)	0 (0)	0 (0)
Total (*n* = 40)	S	**33 (82.5)**	**37 (92.5)**	**40 (100)**	**7 (17.5)**	**21 (52.5)**	**38 (95)**	**5 (12.5)**
	R	**7 (17.5)**	**3 (7.5)**	**0 (0)**	**33 (82.5)**	**19 (47.5)**	**2 (5)**	**35 (87.5)**
Non-ART user								
*E. coli* (*n* = 6)	S	4 (66.7)	4 (66.7)	5 (83.3)	0 (0)	2 (33.3)	6 (100)	1 (16.7)
	R	2 (33.3)	2 (33.3)	1 (16.7)	6 (100)	4 (66.7)	0 (0)	5 (83.3)
*K. rhinose * (*n* = 2)	S	1 (50)	2 (100)	2 (100)	0 (0)	1 (50)	2 (100)	0 (0)
	R	1 (50)	0 (0)	0 (0)	2 (100)	1 (50)	0 (0)	2 (100)
*P. alcalifaciens* (*n* = 1)	S	1 (100)	1 (100)	1 (100)	1 (100)	1 (100)	1 (100)	1 (100)
	R	0 (0)	0 (0)	0 (0)	0 (0)	0 (0)	0 (0)	0 (0)
Total (*n* = 9)	S	**6 (66.7)**	**7 (77.8)**	**8 (88.9)**	**1 (11.1)**	**4 (44.4)**	**9 (100)**	**2 (22.2)**
	R	**3 (33.3)**	**2 (22.2)**	**1 (11.1)**	**8 (88.9)**	**5 (55.6)**	**0 (0)**	**7 (77.7)**

NOR: norfloxacin; C: ceftriaxone; CRO: chloramphenicol; AMP: ampicillin; NA: nalidixic acid; SXT: trimethoprim-sulfamethoxazole; F: nitrofurantoin.

**Table 6:** Antimicrobial sensitivity pattern of Gram positive bacteria isolated from urine culture of ART and non-ART user patients, Jimma University Specialized Hospital, Ethiopia, 2012.

Isolated bacteria	Sensitivity pattern	Antimicrobial agent number (%)							
		NOR	C	CRO	AMP	NA	SXT	E	DA
ART user									
*S. aureus* (*n* = 3)	S	3 (100)	3 (100)	3 (100)	0 (0)	0 (0)	0 (0)	3 (100)	3 (100)
	R	0 (0)	0 (0)	0 (0)	3 (100)	3 (100)	3 (100)	0 (0)	0 (0)
*S. epidermidis* (*n* = 2)	S	2 (100)	2 (100)	2 (100)	1 (50)	0 (0)	0 (0)	2 (100)	2 (100)
	R	0 (0)	0 (0)	0 (0)	1 (50)	2 (100)	2 (100)	0 (0)	0 (0)
*S. saprophyticus* (*n* = 1)	S	1 (100)	1 (100)	1 (100)	0 (0)	0 (0)	0 (0)	1 (100)	1 (100)
	R	0 (0)	0 (0)	0 (0)	1 (100)	1 (100)	1 (100)	0 (0)	0 (0)
Total (*n* = 6)	S	**6 (100)**	**6 (100)**	**6 (100)**	**1 (16.7)**	**0 (0)**	**0 (0)**	**6 (100)**	**6 (100)**
	R	**0 (0)**	**0 (0)**	**0 (0)**	**5 (83.3)**	**6 (100)**	**6 (100)**	**0 (0)**	**0 (0)**
Non-ART user									
*S. epidermidis* (*n* = 1)	S	0 (0)	1 (100)	0 (0)	0 (0)	0 (0)	0 (0)	1 (100)	1 (100)
	R	1 (100)	0 (0)	1 (100)	1 (100)	1 (100)	1 (100)	0 (0)	0 (0)
Total (*n* = 1)	S	**0 (0)**	**1 (100)**	**0 (0)**	**0 (0)**	**0 (0)**	**0 (0)**	**1 (100)**	**1 (100)**
	R	**1 (100)**	**0 (0)**	**1 (100)**	**1 (100)**	**1 (100)**	**1 (100)**	**0 (0)**	**0 (0)**

NOR: norfloxacin; C: ceftriaxone; CRO: chloramphenicol; AMP: ampicillin; NA: nalidixic acid; SXT: trimethoprim-sulfamethoxazole; E: erythromycin; DA: clindamycin.
